# RNF20 Regulates Oocyte Meiotic Spindle Assembly by Recruiting TPM3 to Centromeres and Spindle Poles

**DOI:** 10.1002/advs.202306986

**Published:** 2024-01-19

**Authors:** Liying Wang, Chao Liu, Li Li, Huafang Wei, Wei Wei, Qiuxing Zhou, Yinghong Chen, Tie‐Gang Meng, Renjie Jiao, Zhen‐Bo Wang, Qing‐Yuan Sun, Wei Li

**Affiliations:** ^1^ Guangzhou Women and Children's Medical Center Guangzhou Medical University Guangzhou 510623 China; ^2^ State Key Laboratory of Stem Cell and Reproductive Biology Institute of Zoology Stem Cell and Regenerative Medicine Innovation Institute Chinese Academy of Sciences Beijing 100101 China; ^3^ University of Chinese Academy of Sciences Beijing 100049 China; ^4^ Guangzhou Key Laboratory of Metabolic Diseases and Reproductive Health Guangdong‐Hong Kong Metabolism & Reproduction Joint Laboratory Reproductive Medicine Center Guangdong Second Provincial General Hospital Guangzhou 510317 China; ^5^ The State Key Laboratory of Respiratory Disease Guangzhou Medical University Guangzhou Guangdong 510182 China

**Keywords:** meiosis, oocyte maturation, RNF20, spindle assembly, TPM3

## Abstract

Previously a ring finger protein 20 (RNF20) is found to be essential for meiotic recombination and mediates H2B ubiquitination during spermatogenesis. However, its role in meiotic division is still unknown. Here, it is shown that RNF20 is localized at both centromeres and spindle poles, and it is required for oocyte acentrosomal spindle organization and female fertility. RNF20‐depleted oocytes exhibit severely abnormal spindle and chromosome misalignment caused by defective bipolar organization. Notably, it is found that the function of RNF20 in spindle assembly is not dependent on its E3 ligase activity. Instead, RNF20 regulates spindle assembly by recruiting tropomyosin3 (TPM3) to both centromeres and spindle poles with its coiled‐coil motif. The RNF20‐TPM3 interaction is essential for acentrosomal meiotic spindle assembly. Together, the studies uncover a novel function for RNF20 in mediating TPM3 recruitment to both centromeres and spindle poles during oocyte spindle assembly.

## Graphical abstract

It is shown that RNF20 is a key regulator for acentrosomal spindle assembly and first meiosis completion in oocytes. It is found that the RNF20‐TPM3 interaction is essential for acentrosomal meiotic spindle assembly. RNF20 regulates spindle assembly by recruiting TPM3 to centromeres and spindle poles, which is independent on its E3 ligase activity.

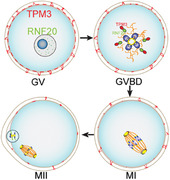

## 1 Introduction

Oocyte meiotic progression is precisely regulated to ensure the production of euploid eggs.^[^ ^1^ ^]^ In the mammalian neonatal ovary, all oocytes are arrested at the diplotene stage of prophase I, characterized by a large nucleus termed a germinal vesicle (GV), and remain arrested at this stage until puberty. In sexually mature individuals, fully grown oocytes remain arrested at the GV stage until luteinizing hormone surges to trigger the resumption of meiosis with GV breakdown (GVBD), followed by dynamic spindle organization and chromosome alignment at prometaphase I (Pro‐MI).^[^ ^2^ ^]^ Uniquely, oocytes use centrosome‐independent pathways to nucleate microtubules for spindle formation and homologous chromosome alignment at the spindle equatorial plane at metaphase I (MI).^[^ ^3^ ^]^ The oocyte then enters anaphase I (AI) and chromosomes start to separate. Subsequently, oocytes extrude the first polar body (PB1) and are arrested at metaphase II (MII) to await fertilization. Meiotic spindles are important structures in the oocytes and are essential indicators of oocyte quality. Abnormal meiotic spindles and disordered chromosome segregation usually result in female infertility.^[^ ^4^ ^]^ Studies have been demonstrated that multiple proteins regulate spindle assembly, including kinesin motors,^[^ ^5^, ^6^ ^]^ centromere‐related proteins,^[^ ^7^, ^8^ ^]^ and various spindle assembly factors.^[^ ^9^ ^]^ However, new factors regulating acentrosomal spindle assembly during oocyte meiosis remain to be explored.

RNF20, in complex with RNF40, was first identified as an E3 ligase for histone H2B monoubiquitination (H2Bub), which is evolutionarily conserved from yeast to mammals.^[^ ^10^, ^11^ ^]^ RNF20 is expressed and works in various tissues and cell types.^[^ ^12^, ^13^, ^14^, ^15^ ^]^ RNF20‐mediated histone H2B monoubiquitination has been shown to regulate DNA replication,^[^ ^16^ ^]^ transcription initiation and elongation,^[^ ^17^, ^18^ ^]^ DNA damage response and repair,^[^ ^19^, ^20^, ^21^ ^]^ nucleosome positioning and occupancy,^[^ ^22^ ^]^ RNA processing and export,^[^ ^23^, ^24^ ^]^ chromosome segregation,^[^ ^25^ ^]^ and maintenance of chromatin boundaries.^[^ ^26^ ^]^ In mice, targeted disruption of RNF20 leads to preimplantation embryonic lethality,^[^ ^27^ ^]^ so we created *Rnf20* flox mice to study its potential function. Using germ cell specific *Rnf20* knockout mice, we found that RNF20‐mediated H2B ubiquitination regulates meiotic recombination by promoting chromatin relaxation during spermatogenesis.^[^ ^15^ ^]^ Previously, it has been reported that RNF20‐mediated ubiquitination is involved in the transcription of some genes in somatic cells.^[^ ^13^, ^28^ ^]^ However, whether RNF20 is involved in meiotic division after meiotic recombination in mammals is unclear. To address this question, we investigated mammalian oocytes, which have completed meiotic recombination and are arrested at the diplotene stage until puberty. In addition, a fully grown oocyte is an excellent model to study transcription‐independent function(s) of RNF20 because transcription is neither active nor required for the two sequential meiotic divisions.^[^ ^29^ ^]^

In this study, we investigated the physiological functions and biochemical mechanisms of RNF20 in regulating mammalian oocyte meiotic maturation. First, we found that RNF20 is localized at centromeres and spindle poles during oocyte meiotic maturation. Next, we found that oocyte‐specific knockout of *Rnf20* results in complete infertility of female mice by causing severe abnormal spindle and chromosome misalignment due to defective bipolar organization of spindles. Furthermore, we demonstrated that RNF20's function in spindle assembly is not dependent on its E3 ligase activity. Instead, the protein recruits TPM3, a tropomyosin family member of actin‐binding proteins, to both centromeres and spindle poles with its coiled‐coil domain. Overall, these results reveal an unknown function of RNF20 during oocyte maturation and clarify mechanisms underlying acentrosomal spindle assembly during meiotic division.

## 2 Results

### 2.1 RNF20 Localizes at Centromeres and Spindle Poles During Mouse Oocyte Meiotic Maturation

**Figure 1:**
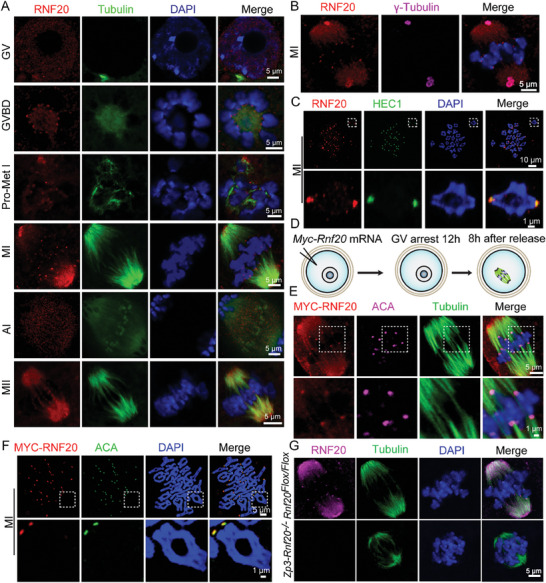
The localization of RNF20 during mouse oocyte meiotic maturation. A) Mouse oocytes at GV, GVBD, Pro‐Met I, MI, AI, and MII stages were immunolabeled with anti‐RNF20 antibody (red), anti‐α‐tubulin antibody (green) and counterstained with 4′,6‐diamidino‐2‐phenylindole (DAPI, blue). B) Immunofluorescence showing the localization of RNF20 (red) in MI stage oocytes. γ‐Tubulin (magenta) is used as a marker for spindle poles. C) Chromosome spreading showing the localization of RNF20 (red) in MI stage oocytes. HEC1 (green) served as a marker for centromere. D) Schematic of *Myc‐Rnf20* mRNA microinjection experiments. E) Mouse oocytes at the GV stage were microinjected with MYC‐RNF20 mRNA and cultured for 8 h. MI stage oocytes were immunolabeled with anti‐MYC antibody (red), anti‐α‐tubulin antibody (green), anti‐centromere antibody (magenta) and counterstained with DAPI (blue). F) Chromosome spreading showing the localization of exogenous RNF20 (red). ACA (green) served as a marker for centromere. G) RNF20 signal disappeared in *Zp3‐Rnf20^−/−^* oocytes. Mouse oocytes at MI stage were immunolabeled with an anti‐RNF20 antibody (magenta), an anti‐α‐tubulin antibody (green), and counterstained with DAPI (blue).

Seeking to better understand the functional roles of RNF20 during mouse oocyte meiotic maturation, we examined the protein's subcellular localization at six sequential maturation stages. Immunofluorescence staining coupled with confocal microscopy showed that RNF20 was predominantly distributed in the nucleus of GV oocytes. Surprisingly, close observation revealed that RNF20 signals accumulated at centromeres and spindle poles from GVBD stage to MI stage. No RNF20 signals were detected at the centromeres in AI stage, but RNF20 signals were again observed at MII stage. To confirm these findings, centromeres and spindle poles were marked with anticentromere antibody (ACA) and γ‐Tubulin, respectively. We found that centromeres and spindle poles indeed colocalized with RNF20 (**Figures** **1A,B**; Figure S1A,B, Supporting Information). Immunostaining of spread chromosomes further confirmed that RNF20 was localized at centromeres (**Figure** **1**C).

To confirm whether the observed RNF20 antibody staining pattern was specific for centromeres and spindle poles, exogenous MYC‐RNF20 was ectopically expressed in mouse oocytes (Figure 1D). We first demonstrated the exogenous MYC‐RNF20 protein was expressed in oocytes without affecting the endogenous RNF20 (Figure S1C, Supporting Information). Immunofluorescent staining confirmed that RNF20 was indeed localized at centromeres and spindle poles during oocyte meiosis (Figure 1E,F). And the centromeres and spindle poles signals disappeared in *Rnf20* knockout oocytes (Figure 1G). These results demonstrate that RNF20 is localized at centromeres and spindle poles during oocyte meiosis, and this localization pattern suggests that RNF20 might participate in oocyte meiotic maturation.

### 2.2 Oocyte‐Specific Knockout of *Rnf20* Results in Complete Female Infertility in Mice

**Figure 2:**
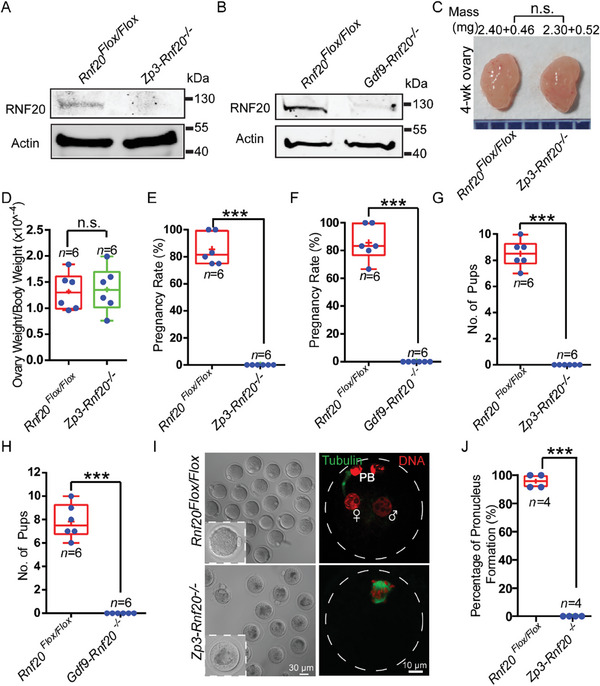
Oocyte‐specific knockout of *Rnf20* results in complete infertility in female mice. A,B) The RNF20 protein level was dramatically reduced in the oocytes of *Zp3‐Rnf20^−/−^* A) and *Gdf9‐Rnf20^−/−^* B) mice. Total proteins from 200 oocytes were loaded in each lane. The blots were probed with anti‐RNF20 and anti‐Actin antibodies. Actin served as a loading control. C) The size of the ovary was consistent in *Zp3‐Rnf20^−/−^* mice compared to the *Rnf20^Flox/Flox^* mice (4‐week‐old, and same as below). D) Quantification ratio of ovary weight/body weight in *Rnf20^Flox/Flox^* and *Zp3‐Rnf20^−/−^* mice. Ovary weight/body weight: *Rnf20^Flox/Flox^*, 1.32 ± 0.15 (n = 6 independent experiments); *Zp3‐Rnf20^−/−^*, 1.36 ± 0.18 (n = 6 independent experiments). Data are presented as mean ± SEM. n.s., non‐significant. Statistical analysis was performed with a two‐tailed unpaired Student's *t*‐test. E,F) *Zp3‐Rnf20^−/−^* and *Gdf9‐Rnf20^−/−^* female mice were completely infertile. Pregnancy rates: *Rnf20^Flox/Flox^*, 85.50% ± 4.75% (n = 6 independent experiments); *Zp3‐Rnf20^−/−^*, 0.00% ± 0.00% (n = 6 independent experiments); *Rnf20^Flox/Flox^*, 85.56% ± 5.21% (n = 6 independent experiments); *Gdf9‐Rnf20^−/−^*, 0.00% ± 0.00% (n = 6 independent experiments); Data are presented as mean ± SEM. ^***^ *p* < 0.001. Statistical analysis was performed with a two‐tailed unpaired Student's *t*‐test. G,H) The average litter size of *Rnf20^Flox/Flox^*, 8.50 ± 0.43 (n = 6 independent experiments); *Zp3‐Rnf20^−/−^*, 0.00% ± 0.00% (n = 6 independent experiments); *Rnf20^Flox/Flox^*, 7.83 ± 0.60 (n = 6 independent experiments); *Gdf9‐Rnf20^−/−^*, 0.00% ± 0.00% (n = 6 independent experiments); Data are presented as mean ± SEM. ^***^ *p* < 0.001. Statistical analysis was performed with a two‐tailed unpaired Student's *t*‐test. I) Pronucleus (PN) formation was blocked in *Zp3‐Rnf20^−/−^* mice. Representative images of embryos derived from *Rnf20^Flox/Flox^* and *Zp3‐Rnf20^−/−^* females. n = 4 mice for zygote stage. Immunofluorescence showing PN formation of *Rnf20^Flox/Flox^* and *Zp3‐Rnf20^−/−^* mice. α‐tubulin (green) and DAPI (red) were co‐stained to show the morphology. Female and male symbols indicate the female and male pronuclei, respectively. PB, polar body. J) Statistical analysis of PN formation in I. *Rnf20^Flox/Flox^*, 95.80% ± 2.43% (n = 4 independent experiments); *Zp3‐Rnf20^−/−^*, 0.00% ± 0.00% (n = 4 independent experiments). Data are presented as mean ± SEM. ^***^ *p* < 0.001. Statistical analysis was performed with a two‐tailed unpaired Student's *t*‐test.

To further investigate the physiological functions of RNF20 during oocyte meiotic maturation, we selectively depleted RNF20 in oocytes by crossing *Rnf20* flox mice with two germ cell specific expressed Cre transgenic mice to generate *Rnf20^Flox/Flox^*; *Zp3‐Cre* and *Rnf20^Flox/Flox^*; *Gdf9‐Cre* (hereafter referred to as *Zp3‐Rnf20^−/−^* and *Gdf9‐Rnf20^−/−^*).^[^ ^15^, ^30^, ^31^ ^]^ *Gdf9‐Cre* is specifically expressed in oocytes starting from primordial follicle stage, at approximately postnatal day 3 (PN3). *Zp3‐Cre* starts to express in oocytes from primary follicle stage after approximately postnatal day 5 (PN5).^[^ ^32^ ^]^ Immunoblotting was used to demonstrate *Rnf20*‐knockout efficiency in *Zp3‐Rnf20^−/−^* and *Gdf9‐Rnf20^−/−^* mice. RNF20 protein levels were dramatically reduced in GV oocytes of *Zp3‐Rnf20^−/−^* and *Gdf9‐Rnf20^−/−^* mice compared with those of *Rnf20^Flox/Flox^* oocytes, suggesting that *Rnf20* was efficiently depleted (**Figure** **2**A,B; Figure S2AB,Supporting Information). No significant differences in the size and weight of the ovary were observed between *Rnf20^Flox/Flox^* and *Zp3‐Rnf20^−/−^* mice (Figure 2C,D), but fertility testing showed that *Zp3‐Rnf20^−/−^* and *Gdf9‐Rnf20^−/−^* female mice were completely infertile (Figure 2E,F). No pups were produced by *Zp3‐Rnf20^−/−^* or *Gdf9‐Rnf20^−/−^* females when crossed with wild‐type (WT) males for at least 6 months (Figure 2G,H). These results demonstrate that oocyte‐specific knockout of *Rnf20* results in complete infertility of female mice, suggesting that *Rnf20* is essential for oogenesis or embryogenesis.

To more fully understand the developmental potential of RNF20‐depleted oocytes, *Rnf20^Flox/Flox^* and *Zp3‐Rnf20^−/−^* female mice were mated with WT males. E2 (embryonic day 2) embryos were then flushed out of the oviducts, and none of the RNF20‐depleted oocytes fertilized normally compared with that of the WT group (Figure S2C,D, Supporting Information). Moreover, no normal pronucleus could be observed in *Zp3‐Rnf20^−/−^* mice (Figure 2I,J). Similar results were found in *Gdf9‐Rnf20^−/−^* female mice (Figure S2E,F, Supporting Information). Furthermore, no overt impact of *Rnf20* knockout on folliculogenesis was detected through hematoxylin‐eosin (H&E) staining. Intact primordial, primary, secondary, and antral follicles were all observed in *Rnf20^Flox/Flox^* and *Zp3‐Rnf20^−/−^* ovaries (Figure S2G,H, Supporting Information), which suggests that female infertility may not be caused by the failure of follicular development but rather by defective oocyte meiotic maturation.

### 2.3 *Rnf20* Knockout Causes Abnormal Spindle Assembly During Meiotic Maturation

**Figure 3:**
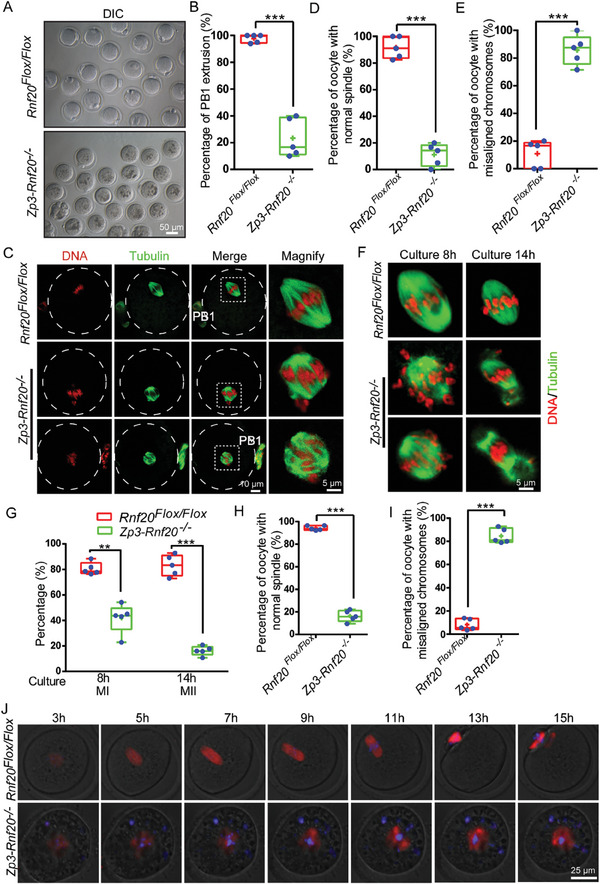
Depletion of RNF20 causes spindle/chromosome abnormalities in mouse oocytes. A) Representative images of oocytes ovulated by 4‐week‐old *Rnf20^Flox/Flox^* and *Zp3‐Rnf20^−/−^* mice after superovulation. B) Quantitative analysis of PB1 extrusion percentage is shown for *Rnf20^Flox/Flox^* and *Zp3‐Rnf20^−/−^* oocytes after superovulation. *Rnf20^Flox/Flox^*, 97.80% ± 1.36% (n = 5 independent experiments, total oocytes = 63); *Zp3‐Rnf20^−/−^*, 23.44% ± 6.45% (n = 5 independent experiments, total oocytes = 68). Data are presented as mean ± SEM. ^***^ *p* < 0.001. Statistical analysis was performed with a two‐tailed unpaired Student's *t*‐test. C) Representative images of spindle morphology and chromosome alignment of *Rnf20^Flox/Flox^* and *Zp3‐Rnf20^−/−^* oocytes in vivo. D) The proportions of normal spindles were recorded for *Rnf20^Flox/Flox^* and *Zp3‐Rnf20^−/−^* oocytes in vivo in C. *Rnf20^Flox/Flox^*, 91.68% ± 3.67% (n = 5 independent experiments, total oocytes = 63); *Zp3‐Rnf20^−/−^*, 11.18% ± 3.75% (n = 5 independent experiments, total oocytes = 68). Data are presented as mean ± SEM. ^***^ *p* < 0.001. Statistical analysis was performed with a two‐tailed unpaired Student's *t*‐test. E) The proportions of misaligned chromosomes were recorded for *Rnf20^Flox/Flox^* and *Zp3‐Rnf20^−/−^* oocytes in vivo in C. *Rnf20^Flox/Flox^*, 10.86% ± 4.47% (n = 5 independent experiments, total oocytes = 63); *Zp3‐Rnf20^−/−^*, 85.80% ± 4.80% (n = 5 independent experiments, total oocytes = 68). Data are presented as mean ± SEM. ^***^ *p* < 0.001. Statistical analysis was performed with a two‐tailed unpaired Student's t‐test. F) Representative images of spindle morphology and chromosome alignment for each genotype at each developmental stage in vitro. G) Developmental rates of cultured oocytes that were collected at the GV stage from *Rnf20^Flox/Flox^* and *Zp3‐Rnf20^−/−^* mice. MI stage: *Rnf20^Flox/Flox^*, 80.54% ± 2.16% (n = 5 independent experiments, total oocytes = 186); *Zp3‐Rnf20^−/−^*, 41.80% ± 5.10% (n = 5 independent experiments, total oocytes = 163). MII stage: *Rnf20^Flox/Flox^*, 83.06% ± 3.68% (n = 5 independent experiments, total oocytes = 195); *Zp3‐Rnf20^−/−^*, 16.22% ± 1.65% (n = 5 independent experiments, total oocytes = 210). Data are presented as mean ± SEM. ^**^ *p* < 0.01 and ^***^ *p* < 0.001. Statistical analysis was performed with a two‐tailed unpaired Student's t‐test. H) The proportions of normal spindles were recorded for *Rnf20^Flox/Flox^* and *Zp3‐Rnf20^−/−^* oocytes after cultured for 8 h in vitro. *Rnf20^Flox/Flox^*, 94.28% ± 0.93% (n = 5 independent experiments, total oocytes = 189); *Zp3‐Rnf20^−/−^*, 16.36% ± 2.23% (n = 5 independent experiments, total oocytes = 127). Data are presented as mean ± SEM. ^***^ *p* < 0.001. Statistical analysis was performed with a two‐tailed unpaired Student's *t*‐test. I) The proportions of misaligned chromosomes were recorded for *Rnf20^Flox/Flox^* and *Zp3‐Rnf20^−/−^* oocytes after cultured 8 h in vitro. *Rnf20^Flox/Flox^*, 8.18% ± 2.21% (n = 5 independent experiments, total oocytes = 106); *Zp3‐Rnf20^−/−^*, 84.54% ± 2.86% (n = 5 independent experiments, total oocytes = 96). Data are presented as mean ± SEM. ^***^ *p* < 0.001. Statistical analysis was performed with a two‐tailed unpaired Student's *t*‐test. J) Live imaging results showing in vitro meiotic division of WT and RNF20‐depleted oocytes. SiR‐tubulin (red) and Hoechst (blue) were co‐stained to show morphology.

Our findings regarding RNF20 localization and female infertility in *Rnf20*‐knockout mice led us to investigate the function of RNF20 in oocyte maturation. Using *Rnf20^Flox/Flox^* and *Zp3‐Rnf20^−/−^* mice receiving superovulation treatment, we collected oocytes from the ampulla of oviducts and analyzed potential defects in oocyte meiotic maturation. We observed that 77% of RNF20‐depleted oocytes failed to emit PB1 and did not go through MI (**Figure** **3**A,B). Immunofluorescence and confocal microscopy showed that the majority of ovulated RNF20‐depleted oocytes contained abnormal spindles and chromosomes that were not properly aligned at the equatorial plate (Figure 3C). Quantitative analysis showed that abnormal spindles and misaligned chromosomes were significantly higher in RNF20‐depleted oocytes than in control oocytes (Figure 3D,E). These results confirm that RNF20 depletion disrupts oocyte meiotic maturation in vivo.

We next isolated fully grown GV oocytes from *Rnf20^Flox/Flox^* and *Zp3‐Rnf20^−/−^* mice and cultured the oocytes in vitro. Consistent with the in vivo results, RNF20‐depleted oocytes showed reduced MI and PB1 emission rates (Figure 3G). Oocytes that resumed meiosis had abnormal spindles and misaligned chromosomes at both the MI and MII stages (Figure 3F). Quantitative analysis also confirmed a higher percentage of abnormal spindles and misaligned chromosomes (Figure 3H,I). We then monitored meiotic maturation of WT and RNF20‐depleted oocytes in vitro using live imaging. Using fluorogenic cell permeable probes SiR‐tubulin and Hoechst, we co‐stained the oocytes to show the spindle assembly and chromosome organization. Compared with WT oocytes, spindle was abnormal, and chromosomes were misaligned at the equatorial plate in RNF20‐depleted oocytes. As previously noted, we found that these oocytes did not emit PB1 and failed to go through MI (Figure 3J). Further analysis showed that conditional knockout of *Rnf20* resulted in collapsed and multipolar spindles (Figure S3A,B, Supporting Information). Collectively, these results demonstrate that conditional knockout of RNF20 affects spindle assembly and chromosome alignment, which further results in the failure of meiosis I completion. This finding fits well with the observation that RNF20 is localized at centromeres and spindle poles during the oocyte meiotic maturation (Figure 1A).

### 2.4 RNF20's Function in Oocyte Is Irrelevant to Its E3 Ligase Activity

**Figure 4:**
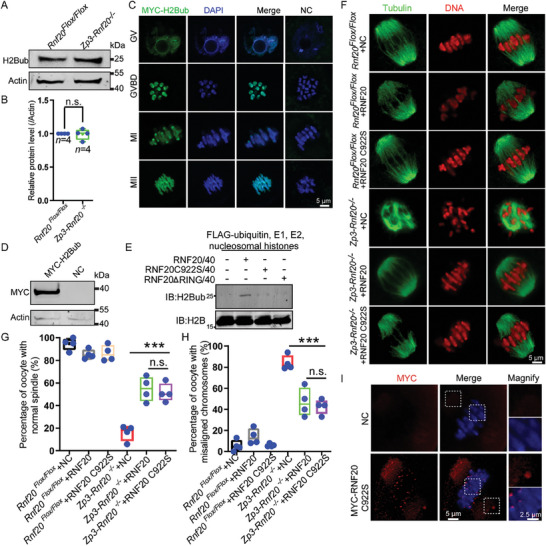
RNF20's function in oocytes is independent of its E3 ligase activity. A) Western blotting showing the expression levels of H2Bub in WT and RNF20‐depleted oocytes. Actin served as a loading control. B) Relative protein levels of H2Bub in *Rnf20^Flox/Flox^* and *Zp3‐Rnf20^−/−^* oocytes. (n = 4 independent experiments). Data are presented as mean ± SEM. n.s., non‐significant. Statistical analysis was performed with a two‐tailed unpaired Student's t‐test. C) Representative images showing H2Bub localization in GV, GVBD, MI, and MII stages during meiosis. Mouse oocytes at GV stage were microinjected with MYC‐H2Bub mRNA and counterstained with anti‐MYC antibody (green) and DAPI (blue). The same amount of RNase‐free water was injected as negative control (NC). D) Western blotting showing protein expression after injecting H2Bub mRNA. Total proteins from 100 injected oocytes were loaded in each lane. The blots were probed with anti‐MYC and anti‐Actin antibodies. E) in vitro ubiquitination assays contained nucleosomal histones, E1, E2, FLAG‐tagged ubiquitin, and RNF20/40 or RNF20 C922S/40, RNF20△RING/40. The blots were probed with anti‐H2B and anti‐H2Bub antibodies. F) Representative images of spindle morphology and chromosome alignment in *Rnf20^Flox/Flox^* + NC, *Rnf20^Flox/Flox^* +RNF20, *Rnf20^Flox/Flox^* +RNF20 C922S, *Zp3‐Rnf20^−/−^* +NC, *Zp3‐Rnf20^−/−^* +RNF20 and *Zp3‐Rnf20^−/−^* +RNF20 C922S. G) The proportions of normal spindles were recorded for the indicated groups. *Rnf20^Flox/Flox^* + NC, 94.50% ± 2.72% (n = 4 independent experiments, total oocytes = 68); *Rnf20^Flox/Flox^* +RNF20, 85.25% ± 2.06% (n = 4 independent experiments, total oocytes = 149); *Rnf20^Flox/Flox^* +RNF20 C922S, 86.23% ± 3.53% (n = 4 independent experiments, total oocytes = 83); *Zp3‐Rnf20^−/−^* +NC, 15.85% ± 3.48% (n = 4 independent experiments, total oocytes = 51); *Zp3‐Rnf20^−/−^* +RNF20, 54.65% ± 5.42% (n = 4 independent experiments, total oocytes = 34); *Zp3‐Rnf20^−/−^* +RNF20 C922S 51.38% ± 4.06% (n = 4 independent experiments, total oocytes = 50). Data are presented as mean ± SEM. ^***^ *p* < 0.001. n.s., non‐significant. Statistical analysis was performed with a two‐tailed unpaired Student's *t*‐test. H) The proportions of misaligned chromosomes were recorded for the indicated groups. *Rnf20^Flox/Flox^* + NC, 5.50% ± 2.72% (n = 4 independent experiments, total oocytes = 68); *Rnf20^Flox/Flox^* +RNF20, 14.25% ± 3.71% (n = 4 independent experiments, total oocytes = 149); *Rnf20^Flox/Flox^* +RNF20 C922S, 6.00% ± 0.71% (n = 4 independent experiments, total oocytes = 83); *Zp3‐Rnf20^−/−^* +NC, 84.00% ± 3.44% (n = 4 independent experiments, total oocytes = 51); *Zp3‐Rnf20^−/−^* +RNF20, 46.75% ± 6.73% (n = 4 independent experiments, total oocytes = 34); *Zp3‐Rnf20^−/−^* +RNF20 C922S 42.70% ± 3.54% (n = 4 independent experiments, total oocytes = 50). Data are presented as mean ± SEM. ^***^ *p* < 0.001. n.s., non‐significant. Statistical analysis was performed with a two‐tailed unpaired Student's *t*‐test. I) Immunofluorescence showing localization of RNF20 C922S. Mouse oocytes at GV stages were microinjected with MYC‐RNF20 C922S mRNA. MI stage oocytes were counterstained with anti‐MYC antibody (red) and DAPI (blue). The same amount of RNase‐free water was injected as negative control. The two small boxes represent the signals of the spindle pole and centromere, respectively.

As an E3 ligase, RNF20 might regulate spindle assembly by targeting specific substrates. Therefore, the identification of RNF20's substrates may provide insight into the protein's physiological functions and mechanisms during oocyte meiotic maturation. H2B is a well‐known substrate of RNF20, and it has been reported that RNF20‐mediated H2B monoubiquitination functions in various cellular processes.^[^ ^33^ ^]^ To our surprise, RNF20 depletion did not affect H2Bub levels in the oocytes (**Figure** **4**A,B). To further elucidate H2Bub's function in oocytes, we next constructed a pCS2plus‐H2Bub plasmid, in which a single ubiquitin molecule was fused to the C‐terminus of H2B via eight glycine residues to enable expression of the fusion protein. After in vitro transcription of the plasmid, the mRNA was then microinjected into GV oocytes to mimic the localization of H2Bub (Figure 4C,D). Immunofluorescence staining showed that H2Bub was predominantly localized in the nucleus of GV oocytes and on chromosomes throughout GVBD to MII stages (Figure 4C), which is different from RNF20 localization. These results indicate that RNF20 might regulate spindle assembly by targeting substrates other than H2Bub during oocyte meiotic maturation.

To further investigate the potential function of RNF20's E3 ligase activity, we created a RING mutant in which the conserved cysteine of the RING‐finger motif was replaced by serine (RNF20 C922S).^[^ ^34^, ^35^ ^]^ We first assessed the E3 ligase activity of RNF20 mutant and confirmed that the E3 ligase activity of RNF20 mutant was lost (Figure 4E). We further evaluated the protein levels for RNF20 mutant in WT oocytes after microinjection and found that the exogenous RNF20 mutant protein was expressed pretty well in oocytes (Figure S4A, Supporting Information). After microinjection of WT RNF20 and RNF20 C922S mRNA into RNF20‐depleted GV stage oocytes, spindle assembly, and chromosome alignment defects were both rescued (Figure 4F–H). Furthermore, RNF20 localization on centromeres and spindle poles was not affected by the disruption of the protein's E3 ligase activity (Figure 4I). These results indicate that the spindle assembly‐related, meiosis‐specific functions of RNF20 are not dependent on its E3 ligase activity.

### 2.5 RNF20 Recruits TPM3 to Centromeres and Spindle Poles

Recently, In et al. (2019) identified 598 potential RNF20‐interacting proteins via mass spectrometry.^[^ ^36^ ^]^ Among these proteins, we found the following proteins (RBBP4, RBBP7, and TPM3) are likely participate in oocyte meiosis. Previous studies indicate that both RBBP4 and RBBP7 are required for spindle assembly and chromosome alignment during oocyte meiotic maturation.^[^ ^37^, ^38^ ^]^ TPM3 is thought to be involved in oocyte meiotic maturation.^[^ ^13^, ^39^ ^]^ As a RNF20 potential partner, ubiquitin associated and SH3 domain‐containing B (UBASH3B) mediated proper targeting of Aurora B to microtubules, and it is essential for ensuring accurate timing and chromosome segregation fidelity during mitosis.^[^ ^40^ ^]^ Therefore, all these four candidates were selected for further studies.

**Figure 5:**
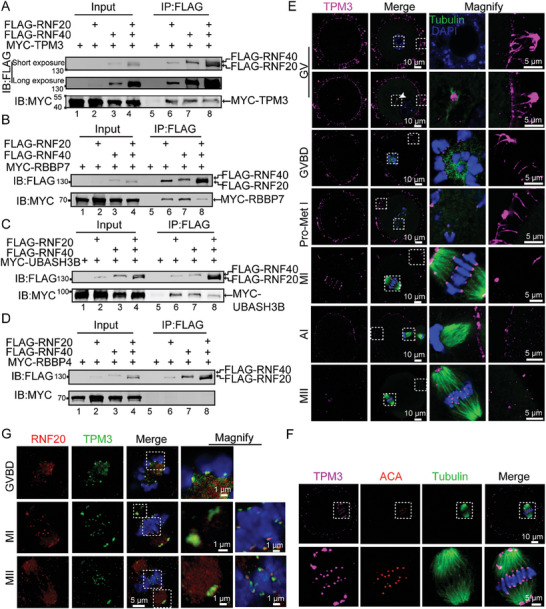
RNF20 is co‐localized with TPM3 in spindle poles and centromeres during meiosis. A–D) Co‐IP results show intracellular interaction of RNF20 with TPM3 (A), RBBP7 (B) and UBASH3B (C), but not RBBP4 D). HEK293T cells were transiently co‐transfected with vectors expressing FLAG‐RNF20, FLAG‐RNF40, and MYC‐TPM3, MYC‐RBBP7, MYC‐UBASH3B, or MYC‐RBBP4. Whole‐cell lysate was subjected to immunoprecipitation with protein A beads and visualized by Western blotting using the indicated antibodies. E) Mouse oocytes at GV, GVBD, Pro‐Met I, MI, AI, and MII stages were immunolabeled with anti‐TPM3 antibody (magenta), anti‐α‐tubulin antibody (green) and counterstained with DAPI (blue). The first set of magnified columns represents the signals of TPM3 at the spindle pole and centromere, while the second set of magnified columns represents the signals of TPM3 at the cortex. F) Immunofluorescence showing localization of TPM3 (magenta). ACA (red) served as a marker for centromere. G) Co‐localization of endogenous RNF20 with TPM3 during different phases of meiosis in oocytes. The distribution of endogenous RNF20 and TPM3 was detected using immunofluorescence assays with anti‐RNF20 (red) and anti‐TPM3 antibodies (green).

To test the relationship between RNF20 and these candidate oocyte‐maturation proteins, we construct plasmids MYC‐TPM3, MYC‐UBASH3B, MYC‐RBBP4, MYC‐RBBP7, FLAG‐RNF20, and FLAG‐RNF40. The plasmids of the four potential interacting proteins were co‐transfected into HEK293T cells with RNF20/RNF40 by lipofectamine 2000 reagent. Western blotting results showed that all these genes were successfully expressed in HEK293T cells (Figure S4B–G, Supporting Information). Co‐immunoprecipitation (Co‐IP) analysis revealed that TPM3, UBASH3B and RBBP7, but not RBBP4 indeed could interact with RNF20, RNF40, and the RNF20/RNF40 complex (**Figure** **5**A–D). Thus, TPM3, UBASH3B, and RBBP7 may be involved in oocyte maturation.

**Figure 6:**
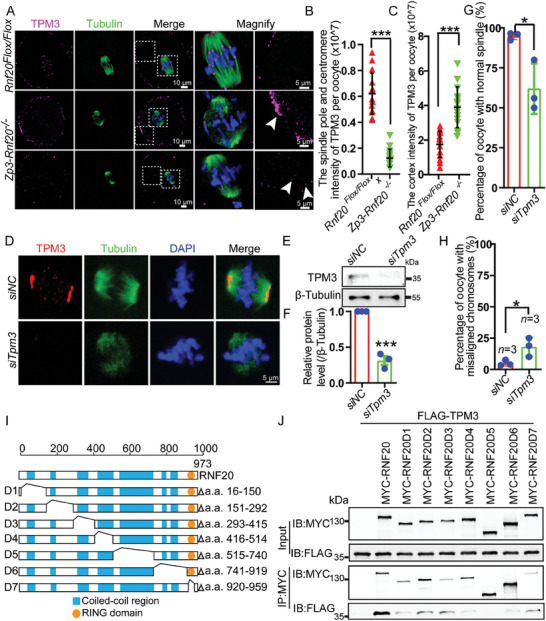
Mapping the interaction regions of TPM3 and RNF20. A) Representative images showing TPM3 localization in WT and RNF20‐depleted oocytes during the indicated developmental stages. Arrows indicate TPM3 signals in the cortex or cytoplasm. B) Statistical analysis of the spindle pole and centromere signal of TPM3 per oocyte at MI stage. *Rnf20^Flox/Flox^*, 0.62 ± 0.16 (n = 15); *Zp3‐Rnf20^−/−^*, 0.12 ± 0.07 (n = 15). Data are presented as mean ± SD. ^***^ *p* < 0.001. n indicates a number of oocytes analyzed. Statistical analysis was performed with a two‐tailed unpaired Student's *t*‐test. C) Statistical analysis of the cortex signal of TPM3 per oocyte at MI stage. *Rnf20^Flox/Flox^*, 1.83 ± 0.76 (n = 15); *Zp3‐Rnf20^−/−^*, 3.98 ± 1.18 (n = 15). Data are presented as mean ± SD. ^***^ *p* < 0.001. n indicates a number of oocytes analyzed. Statistical analysis was performed with a two‐tailed unpaired Student's *t*‐test. D) Representative images of spindle morphology in *siNC* or *siTpm3* groups oocytes. E) Western blotting showing the expression level of TPM3 in siNC or siTpm3 group oocytes. β‐Tubulin served as a loading control. F) Relative protein level of TPM3 in siNC or siTpm3 group oocytes. (n = 3 independent experiments). Data are presented as mean ± SEM. ^***^ *p* < 0.001. Statistical analysis was performed with a two‐tailed unpaired Student's *t*‐test. G) The proportion of normal spindles was recorded for *siNC* or *siTpm3* groups oocytes. *siNC*, 94.93% ± 1.33% (n = 3 independent experiments, total oocytes = 69); *siTpm3*, 62.00% ± 9.17% (n = 3 independent experiments, total oocytes = 110). Data are presented as mean ± SEM. ^*^ *p* < 0.05. Statistical analysis was performed with a two‐tailed unpaired Student's *t*‐test. H) The proportion of misaligned chromosomes was recorded for *siNC* or *siTpm3* groups oocytes. *siNC*, 5.07% ± 1.33% (n = 3 independent experiments, total oocytes = 69); *siTpm3*, 18.00% ± 4.36% (n = 3 independent experiments, total oocytes = 110). Data are presented as mean ± SEM. ^*^ *p* < 0.05. Statistical analysis was performed with a two‐tailed unpaired Student's *t*‐test. I) Schematic of the domain architecture of RNF20 and its deletion mutants which were used in this study. J) A coiled‐coil motif of RNF20 interacts with TPM3. MYC‐tagged wild‐type RNF20 and its internal deletion mutants were expressed in HEK293T cells. Whole‐cell lysate was subjected to immunoprecipitation with protein A beads and visualized by Western blotting using the indicated antibodies.

To further test the relationship between RNF20 and TPM3, UBASH3B, or RBBP7, we examined the localization of TPM3, UBASH3B, and RBBP7 during oocyte meiotic maturation. RBBP7 was localized in the nucleus of GV stage oocytes, while no RBBP7 signal was observed at the GVBD or MI stage (Figure S4H, Supporting Information). UBASH3B was localized in the nucleus of GV stage oocytes, and spindle poles of MI and MII oocytes (Figure S4I, Supporting Information). The localization of UBASH3B or RBBP7 was different from that of RNF20. TPM3 signals accumulated at the cortex, centromeres, and spindle poles throughout GVBD to MI stages. TPM3 was localized only at the cortex during AI stage but was again observed at centromeres and spindle poles at the MII stage (Figure 5E,F). Additionally, we confirmed the co‐localization of TPM3 with RNF20 at centromeres and spindle poles from GVBD to MI stages (Figure 5G). Chromosome spread analyses also revealed the co‐localization of TPM3 with RNF20 at centromeres (Figure S5A, Supporting Information). Furthermore, immunofluorescence staining results demonstrated that RNF20 depletion had no effect on the expression and localization of RBBP7 and UBASH3B (Figure S4J,K, Supporting Information), while the localization of TPM3 was affected clearly (**Figure** **6**A). This suggests that RNF20 might regulate spindle assembly by interacting with TPM3.

Recalling our observation that RNF20 depletion has no effect on H2Bub, RNF20 interacts and co‐localizes with TPM3 at centromeres and spindle poles in the oocytes, and depletion of RNF20 affected the localization of TPM3 (Figure 6A), we postulated that the meiosis‐specific function of RNF20 may be recruiting TPM3 to both centromeres and spindle poles.

### 2.6 RNF20 Incorporates with TPM3 to Regulate Spindle Assembly During Oocyte Maturation

To further study the relationship between RNF20 and TPM3 during oocyte maturation, we investigated TPM3 in RNF20‐depleted oocytes. Although RNF20 depletion did not affect the expression level of TPM3 (Figure S5B,C, Supporting Information), immunofluorescence staining showed an absence of TPM3 signals at centromeres and spindle poles in RNF20‐depleted oocytes and strong signals in WT oocytes (Figure 6A). Further quantification of TPM3 signals in RNF20‐depleted oocytes indicated that the cortex intensity of TPM3 was significantly increased, and the spindle pole and centromere intensity of TPM3 was decreased compared with that of WT oocytes (Figure 6B,C), suggesting the depletion of RNF20 resulted in inadequate recruitment of TPM3 to spindle poles and centromeres. In addition, we used small interfering RNA (*siRNA*) *(non‐coding [NC] or Tpm3*) in WT oocytes to evaluate the consequences of *Tpm3* knockdown on oocyte meiotic maturation. We found that *Tpm3* knockdown led to abnormal spindles and misaligned chromosomes at MI stage (Figure 6D–F). Quantitative analysis showed a significantly higher percentage of abnormal spindles and misaligned chromosomes in *siTmp3*‐treated oocytes compared with *siNC*‐treated oocytes (Figure 6G,H), which is similar to outcomes with RNF20 depletion. Together, these results suggest that RNF20 may interact with TPM3 to regulate spindle assembly during oocyte maturation.

### 2.7 TPM3 Interacts with the Coiled‐Coil Domain of RNF20

The 3D structure of RNF20 shows that RNF20 contains seven coiled‐coil domains and a Ring domain, which are all evolutionarily conserved from yeast to mammals.^[^ ^11^ ^]^ To determine any interaction(s) between RNF20 and TPM3, we generated a series of internal deletion mutants of *Rnf20*, D1‐D7 (Figure 6I). These mutants, together with TPM3, were transfected into HEK293T cells using lipofectamine 2000 reagent. After immunoprecipitation with an anti‐MYC antibody, we found that only the D5 mutant of RNF20 abolished the interactions between TPM3 and RNF20 (Figure 6J). Since D5 lacks the coiled‐coil domain, it is likely the coiled‐coil domain of RNF20 is needed to recognize and interact with TPM3.

### 2.8 The Interaction Between RNF20 and TPM3 Is Essential for Spindle Assembly During Oocyte Maturation

**Figure 7:**
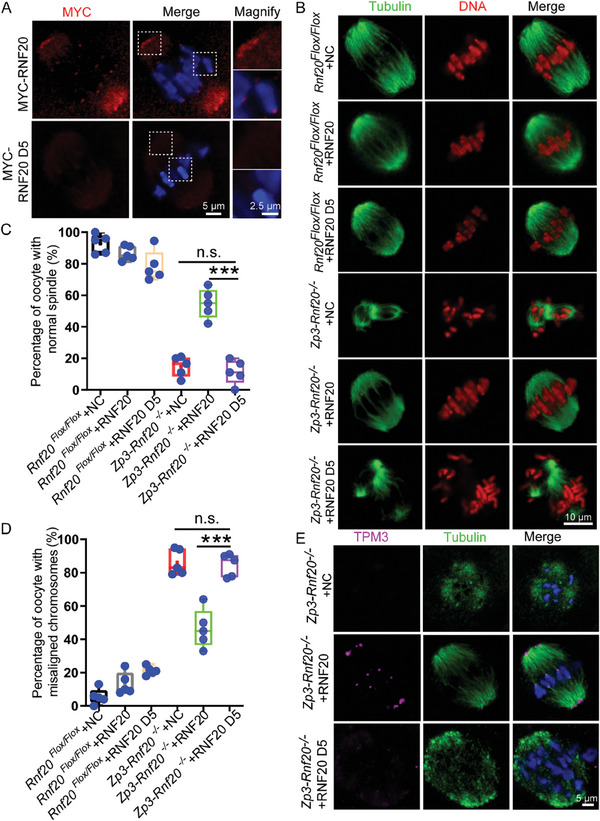
TPM3 is recruited to spindle poles and centromeres through the coiled‐coil domain of RNF20. A) Immunofluorescence showing localization of RNF20 and RNF20 D5. Mouse oocytes at GV stages were microinjected with MYC‐RNF20 mRNA and MYC‐RNF20 D5 mRNA and cultured for 8 h. MI stage oocytes were counterstained with anti‐MYC antibody (red) and DAPI (blue). The two small boxes represent the signals of the spindle pole and centromere, respectively. B) Representative images of spindle morphology and chromosome alignment in *Rnf20^Flox/Flox^* +NC, *Rnf20^Flox/Flox^* +RNF20, *Rnf20^Flox/Flox^* +RNF20 D5, *Zp3‐Rnf20^−/−^* +NC, *Zp3‐Rnf20^−/−^* +RNF20 and *Zp3‐Rnf20^−/−^* +RNF20 D5 oocytes. C) The proportions of normal spindles were recorded for the indicated groups. *Rnf20^Flox/Flox^* +NC, 92.80% ± 2.71% (n = 5 independent experiments, total oocytes = 80); *Rnf20^Flox/Flox^* +RNF20, 86.60% ± 2.09% (n = 5 independent experiments, total oocytes = 161); *Rnf20^Flox/Flox^* +RNF20 D5, 78.50% ± 4.32% (n = 5 independent experiments, total oocytes = 73); *Zp3‐Rnf20^−/−^* +NC, 14.88% ± 2.87% (n = 5 independent experiments, total oocytes = 63); *Zp3‐Rnf20^−/−^* +RNF20, 54.72% ± 4.20% (n = 5 independent experiments, total oocytes = 45); *Zp3‐Rnf20^−/−^* +RNF20 D5, 11.38% ± 3.45% (n = 5 independent experiments, total oocytes = 70). Data are presented as mean ± SEM. ^***^ *p* < 0.001. n.s., non‐significant. Statistical analysis was performed with a two‐tailed unpaired Student's *t*‐test. D) The proportions of misaligned chromosomes were recorded for the indicated groups. *Rnf20^Flox/Flox^* +NC, 5.60% ± 2.11% (n = 5 independent experiments, total oocytes = 80); *Rnf20^Flox/Flox^* +RNF20, 13.40% ± 2.99% (n = 5 independent experiments, total oocytes = 161); *Rnf20^Flox/Flox^* +RNF20 D5, 21.20% ± 1.16% (n = 5 independent experiments, total oocytes = 73); *Zp3‐Rnf20^−/−^* +NC, 86.20% ± 3.46% (n = 5 independent experiments, total oocytes = 63); *Zp3‐Rnf20^−/−^* +RNF20, 46.40% ± 5.22% (n = 5 independent experiments, total oocytes = 45); *Zp3‐Rnf20^−/−^* +RNF20 D5, 84.60% ± 3.06% (n = 5 independent experiments, total oocytes = 70). Data are presented as mean ± SEM. ^***^ *p* < 0.001. n.s., non‐significant. Statistical analysis was performed with a two‐tailed unpaired Student's *t*‐test. E) Representative images showing TPM3 localization in *Zp3‐Rnf20^−/−^* +NC, *Zp3‐Rnf20^−/−^* +RNF20 and *Zp3‐Rnf20^−/−^* +RNF20 D5 oocytes.

To further characterize the D5 mutant of *Rnf20*, we microinjected WT RNF20 and D5 mRNA into mouse oocytes. In RNF20 D5‐mutant expressing oocytes, we found RNF20 signals disappeared at centromeres and spindle poles (**Figure** **7**A; Figure S4A, Supporting Information), suggesting the fifth coiled‐coil domain is important for the localization of RNF20.

To further confirm whether RNF20 regulates spindle assembly and chromosome alignment through the interaction of TPM3 and the coiled‐coil domain of RNF20, we detected and quantified the percentage of oocytes with abnormal spindles and misaligned chromosomes after expressing the mutant in RNF20‐depleted oocytes. We found that the spindle assembly and chromosome alignment defects were partially rescued by complementing with WT RNF20, but not RNF20 D5 (Figure 7B–D). These results support that the coiled‐coil domain of RNF20 is required for spindle assembly and chromosome alignment. Moreover, we confirmed that WT RNF20, but not RNF20 D5, could rescue the localization of TPM3 at spindle poles and centromeres in RNF20‐depleted oocytes (Figure 7E), indicating that TPM3 is recruited to spindle poles and centromeres through the fifth coiled‐coil domain of RNF20. Collectively, these results demonstrate that RNF20 regulates spindle assembly and chromosome alignment by recruiting TPM3 to spindle poles and centromeres.

## 3 Discussion

**Figure 8:**
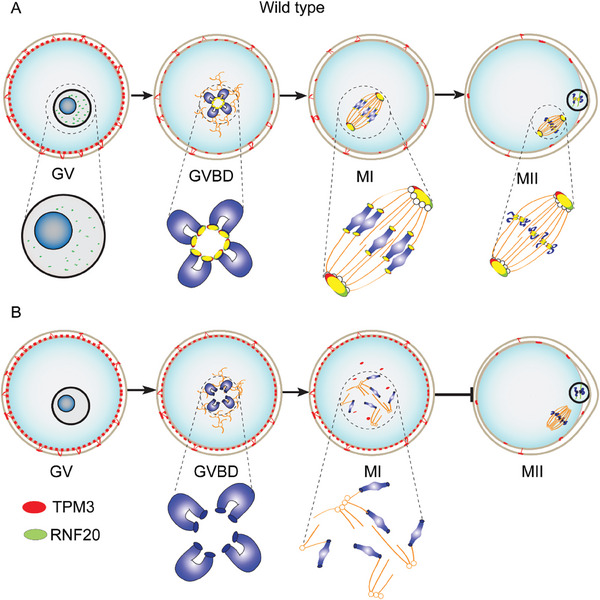
Proposed model for the function of RNF20 during oocyte meiotic maturation. A) In GV oocytes, RNF20 and TPM3 are localized in the nucleus and cortex of oocyte, respectively. When the germinal vesicle breaks down, TPM3 is recruited to centromeres through the coiled‐coil motif of RNF20. Subsequently, RNF20 and TPM3 accumulate in spindly poles and centromeres, coordinating with other spindle assembly‐associated proteins to regulate spindly assembly, forming a bipolar spindle by metaphase I and finally finishing the entire oocyte meiotic maturation. B) In the RNF20‐deficient oocytes, TPM3 cannot be recruited to spindly poles and centromeres. As such, spindle was abnormal and chromosomes are misaligned. Thereafter, these RNF20‐depleted oocytes do not complete meiosis I and fail to emit PB1, ultimately resulting in female infertility in mice.

Previously, we generated *Rnf20* flox mice, by crossing with *Stra8* transgenic mice, we show that *Rnf20* is essential for male fertility. We also showed RNF20‐mediated H2B ubiquitination participates in meiotic recombination by promoting chromatin relaxing, thus facilitating DNA repair factors to be recruited to DSB sites.^[^ ^15^ ^]^ However, whether RNF20 has other functions during meiotic division remained unknown. Additionally, oocyte meiosis progression is different from that of male germ cells in several aspects, including long‐term arrest at the prophase stage of meiosis I, spindle assembly in the absence of centrosomes, and error‐prone chromosome separation. The mechanisms underlying acentrosomal spindle organization and the production of euploid oocytes are far from well understood. Here, we show that RNF20 is a key regulator of acentrosomal spindle assembly and first meiosis completion of oocytes. RNF20 regulates spindle assembly by recruiting TPM3 to centromeres and spindle poles (**Figure** **8**).

Numerous studies have shown that ubiquitin plays a crucial role in regulating spindle assembly in various species. It has been implicated in regulating different aspects of spindle assembly, including centrosome clustering, spindle pole functions, microtubule assembly and polymerization, microtubule motors, and other essential processes.^[^ ^41^, ^42^, ^43^, ^44^ ^]^ SCF^Cdc4^ ubiquitin ligase regulates spindle pole separation and the assembly of a bipolar spindle in yeast.^[^ ^45^ ^]^ In *Drosophila*, *C. elegans*, and human cells, the SCF‐Slimb/β‐TRCP complex targets *Plk4* to regulate centriole assembly.^[^ ^46^, ^47^, ^48^, ^49^ ^]^ Certain TRIM proteins, such as TRIM28 and TRIM19 localize at centrosomes and spindle poles to regulate the appropriate number of centrosomes in mammalian cells.^[^ ^50^, ^51^ ^]^ TRIM69A and TRIM22 are essential for centrosome clustering,^[^ ^52^, ^53^ ^]^ while TRIM69 and TRIM36 ensure proper attachment of microtubules to kinetochores in mammalian cells.^[^ ^53^, ^54^ ^]^ Additionally, ubiquitin E3 ligases like MGRN1, VHL, Xnf7 (*Xenopus* nuclear factor 7), and SIAH1 have been found to participate in spindle assembly by regulating microtubule assembly or polymerization.^[^ ^55^, ^56^, ^57^, ^58^ ^]^ Besides ubiquitin ligases, the deubiquitinating enzyme BRCC36 isopeptidase complex (BRISC), cylindromatosis (CYLD), and USP11 also play a significant role in spindle assembly by modulating bipolar spindle formation.^[^ ^59^, ^60^, ^61^ ^]^

RNF20 is evolutionarily conserved from yeast to mammals, and it is widely associated with the cell cycle, stem cell differentiation, development, tumorigenesis, and apoptosis,^[^ ^12^, ^18^, ^62^, ^63^, ^64^ ^]^ with a majority of its functions coming from its ability to mediate H2B ubiquitination. Here, for the first time, we showed that RNF20 is localized at centromeres and spindle poles, and it is required for spindle assembly during oocyte meiotic division. In sharp contrast to the previous reports about the function of ubiquitin in spindle assembly, we found that RNF20's function in oocyte spindle assembly is not dependent on its E3 ligase activity, suggesting that this protein may have evolved a novel function for this specific process. Furthermore, we show that RNF20 colocalizes and interacts with TPM3 on spindle poles and centromeres. Importantly, the interaction between these two proteins is essential for spindle assembly during oocyte meiotic divisions, thus assigning a new function to RNF20 during oocyte meiotic divisions.

Tropomyosin is a canonical coiled‐coil protein that regulates actin filament functions in muscle and non‐muscle cells.^[^ ^65^ ^]^ The mammalian Tm family consists of four genes: αTm, βTm, γTm, and δTm (*Tpm1‐4*). TPM3 produces at least 11 distinct cytoskeletal isoforms via alternative splicing.^[^ ^66^, ^67^ ^]^ These isoforms take part in various cellular processes, including cell trafficking and cytokinesis, morphogenesis, and cell proliferation.^[^ ^68^, ^69^, ^70^ ^]^ Previous studies have reported that TPM3 knockout results in embryo lethality.^[^ ^71^ ^]^ However, the regulatory mechanisms of *Tpm3* during oocyte meiotic maturation are not fully understood. Jang et al. (2014) studied the potential functions of TPM3 by injecting *siRNA* into oocytes to knockdown its expression,^[^ ^39^ ^]^ and found that TPM3 is localized in the oocyte cortex at the GV stage and participates in asymmetric cell division and maintenance of cortical integrity. We observed TPM3 localization in centromeres, spindle poles, and cortex of oocytes at different stages. It is required for spindle assembly and chromosome alignment at MI stage of oocytes (Figures 5E and 6D). The differences might occur for several reasons. First, after GVBD, TPM3 is redistributed to centromeres and spindle poles, but it needs a higher magnification to clearly show specific localization. Alternatively, our *siRNA* targets transcript variant 1 of *Tpm3* coding DNA sequence (CDS), which is the longest transcript variant of TPM3, but their targeted site is located downstream (about 500 bp) of transcript variant 3. Additionally, their knockdown efficiency is only validated by real‐time PCR, while our knockdown efficiency is validated by immunofluorescence and Western blotting of TPM3 in the oocytes (Figure 6D,G,H). Finally, the so‐called symmetrical division phenotype has never been validated by in vivo studies (such as *Cdc42*).^[^ ^72^ ^]^ All these possibilities still need further experimental validation, and the construct of an oocyte specific *Tpm3* knockout model should be a nice way to understand the detailed physiological functions of TPM3 during oocyte meiotic maturation.

The formation of a bipolar spindle structure requires the coordination of the motor dynein, nucleators, kinases, phosphatase, other microtubule‐associated proteins and various spindle assembly factors.^[^ ^73^, ^74^, ^75^ ^]^ Kinetochore mainly functions in establishing proper microtubule–kinetochore attachments. Additionally, centromeres are the specific chromosomal regions that serve as the platform for kinetochores to assemble.^[^ ^76^, ^77^ ^]^ Both coordinately contribute to spindle assembly, but they usually assemble by different mechanisms, few factors are found to participate in these two processes at the same time. In our studies, we found that RNF20 and TPM3 localized at centromeres and spindle poles simultaneously. They play a crucial role in meiotic spindle assembly and chromosome alignment. We observed that the depletion of RNF20 leads to the formation of collapsed and multipolar spindles (Figure S3A,B, Supporting Information). This phenomenon may arise from RNF20's role in regulating the convergence of centriolar microtubule‐organizing centers (aMTOCs) and microtubule nucleation at the spindle pole. Furthermore, we found that RNF20‐depleted oocytes exhibit chromosome misalignment and arrest at the MI stage (Figure 3C,F). This could be attributed to the localization of RNF20/TPM3 at the centromere and its involvement in the regulation of microtubule‐kinetochore attachment, influencing chromosome segregation during this process. In addition to the known components of acentrosomal spindle poles and centromeres, there may be unidentified proteins that contribute to meiotic spindle assembly and chromosome alignment. Therefore, it is worthwhile to investigate the specific components of acentrosomal spindle poles and centromeres in future research. Furthermore, according to our studies, RNF20 recruits TPM3 to both centromeres and spindle poles with its coiled‐coil motif. Future studies will need to identify partner proteins of RNF20 and TPM3 on spindle poles and/or kinetochores. Detailed structural studies of these proteins will deepen our understanding of the basic process of meiotic spindle assembly and may provide potential targets for the development of therapies to treat female infertility that is related to disrupted oocyte meiotic maturation.

## 4 Experimental Section

### Animals

The *Rnf20* ^*flox/flox*^ mice were previously generated.^[^ ^15^ ^]^ Briefly, the LoxP sites were inserted into the *Rnf20* genomic region, which includes exons 2, 3, and 4, using recombineering to generate *Rnf20* ^flox/+^ mice. The mice were maintained with a B6/129 genetic background. Mice that lacked *Rnf20* in their oocytes were generated by crossing *Rnf20* ^*flox/flox*^ mice with *Zp3* promoter‐mediated Cre transgenic mice or *Gdf9* promoter‐mediated Cre transgenic mice. Mice were maintained under specific pathogen‐free (SPF) conditions in a controlled environment with temperatures of 20–22 °C, with light/dark cycles of 12/12 h, with a humidity of 50–70%, and food and water provided ad libitum. All animal experiments were performed according to approved institutional animal care and use committee (IACUC) protocols (#2021‐002) of the Institute of Zoology, Chinese Academy of Sciences.

### Antibodies and Proteins

Mouse antibody to α‐Tubulin‐FITC (1:400, F2168) was purchased from Sigma (USA). Mouse antibodies to γ‐Tubulin (1:200, ab11316) and TPM3 (1:100, ab113692) were purchased from Abcam (USA). Mouse antibody to FLAG (1:2000, M20008L) was purchased from Abmart (Shanghai, China). Rabbit antibody to MYC (1:1000, BE2011) was purchased from EASYBIO (Beijing, China). Rabbit antibodies to β‐Actin (1:2000, AC026) and β‐Tubulin (1:1000, AC008) were purchased from ABclonal (Wuhan, China). Rabbit antibodies to RNF20 (1: 1000, 21625‐1‐AP) and TPM3 (1:1000, 10737‐1‐AP) were purchased from Proteintech Group (USA). Rabbit antibodies to H2Bub (1: 1000, 5546s), H2B (1: 1000, 12364S) and H3 (1: 1000, 4499S) were purchased from Cell Signaling Technology (USA). Mouse antibody to HEC1 (1: 100, sc‐515510) was purchased from Santa Cruz Biotechnology (USA). Rabbit antibodies to RBBP7 (1:100, bs8620) and UBASH3B (1:100, bs8741) were purchased from Bioworld (Beijing, China). Human antibody to ACA (1: 200, 15–234) was purchased from Antibodiesinc (USA). Goat anti‐rabbit FITC (1:200, ZF‐0311) and goat anti‐mouse TRITC (1:200, ZB‐2305) were purchased from ZSGB‐BIO (Beijing, China). Alexa Fluor 680‐conjugated goat anti‐mouse (1:10000, A21057) and Alexa Fluor 800‐conjugated goat anti‐rabbit (1:10000, A21109) were purchased from Invitrogen (USA).

### Oocyte Collection and Culture

Female mice (4‐week‐old) were injected with 5 IU of pregnant mare serum gonadotrophin (PMSG, 110254564, Ningbo Second Hormone Company) and humanely euthanized 46 h later. The GV oocytes used in the study were collected from ovaries and placed in M2 medium (M7167, Sigma–Aldrich). These oocytes were then cultured in mini‐drops and covered with mineral oil (M5310, Sigma–Aldrich) left to mature at 37 °C in a 5% CO_2_ atmosphere for in vitro maturation.

### Superovulation and Fertilization

For superovulation, female mice were injected intraperitoneally with 5 IU PMSG followed 46–48 h later with 5 IU human chorionic gonadotropins (hCG, 110251281, Ningbo Second Hormone Company). After an additional 13 h, oocyte/cumulus masses were surgically removed from the ampulla of oviducts, and the oocytes were harvested and used for the subsequent experiments after digestion with 0.3% hyaluronidase (Sigma‐Aldrich) to remove cumulus cells. To obtain fertilized eggs (zygotes), 8‐week‐old female mice were mated with 10‐week‐old WT males. Successful mating was confirmed by the presence of vaginal plugs. E0.5 (zygotes) embryos and E2 (2‐cell to 4‐cell) embryos were flushed out of the oviducts and used for immunofluorescent assay.

### Histological Analysis

Mouse ovaries were fixed in 4% paraformaldehyde (PFA, P1110, Solarbio, China) overnight at 4 °C, dehydrated in an ethanol series and embedded in paraffin. Then, 5‐um sections were cut with a microtome. Following deparaffinization and rehydration, the sections were stained with hematoxylin and eosin (G1100, Solarbio, China) to observe follicle development. Images were collected with a Nikon inverted microscope with a charge coupled device (CCD) (Nikon, Eclipse Ti‐S, Tokyo, Japan).

### Confocal Microscopy for Mouse Oocytes

Oocytes were fixed and permeabilized in 4% PFA and 0.5% Triton X‐100 for 20 min at room temperature. After blocking with 1% bovine serum albumin (BSA, AP0027, Amresco, USA) in PBS, oocytes were incubated with primary antibodies diluted in 1% BSA overnight at 4 °C. Following three washes with PBS, oocytes were labelled with secondary antibodies for 1 h at 37 °C, and then stained with 4′,6‐diamidino‐2‐phenylindole (DAPI) for 5 min. Finally, oocytes were mounted on glass slides and observed with an LSM 780 microscope (Zeiss, Oberkochen, Germany) or SP8 microscope (Leica, Wetzlar, Germany).

### Chromosome Spreading

Oocytes were collected and the zona pellucida was removed using Tyrode's solution (T1788, Sigma–Aldrich), and then the oocytes were dispersed on a coverslip that contained the fixation solution (1% PFA, 3 mm dithiothreitol, and 0.15% Triton X‐100, pH 9.2) for 1 h and air dried. Immunofluorescent staining was performed as in oocytes described above.

### Fertility Testing

For fertility testing, 4–6 individually *Rnf20* ^*flox/flox*^ and *Gdf9‐Rnf20* ^*−/−*^ or *Zp3‐Rnf20* ^*−/−*^ female mice at the age of 8 weeks were continuously mated with 10 weeks old fertile males, and their vaginal plugs were checked every morning. The plugged females were separated and caged individually; the pregnancy outcomes were then recorded. If a female did not miscarry within 22 days post‐mating or did not give birth to offspring by 22 days post‐mating, they were considered to be not pregnant. The numbers of pups and litters were recorded for up to 6 months.

### In Vitro Transcription, Microinjection, and Knockdown Experiment

Capped cRNA was synthesized using the SP6 mMESSAGE mMACHINE Kit (AM1340; Invitrogen) and purified using the RNeasy Micro Kit (Qiagen) according to the manufacturer's instructions. For microinjection, 0.5‐1.0 µg µl^−1^ mRNA was injected into the cytoplasm of oocytes and arrested at the GV stage with 200 µm 3‐isobutyl‐1‐methylxanthine (IBMX, I5897, Sigma–Aldrich) for 12 h, allowing enough time for translation, followed by releasing into IBMX‐free M2 medium for further study. The same amount of RNase‐free water was injected as negative control (NC).

For knockdown experiments, *siRNAs* of TPM3 (Sangon Biotech) were microinjected into the cytoplasm to deplete TPM3. The subsequent *siRNAs* were used at 50 µm, TPM3 *siRNA*‐1: (sense: 5′‐GCUGGACCUGAACGAGAUGUATT‐3′; anUisense: 5′‐UACAUCUCGUUCAGGUCCAGCTT‐3′), TPM3 *siRNA*‐2: (sense: 5′‐ CAGAACCUGAAGUGUCUGAGUTT‐3′; anUisense: 5′‐ ACUCAGACACUUCAGGUUCUGTT‐3′), TPM3 *siRNA*‐3: (sense: 5′‐ GAGCUGGACAAGUAUUCGGAATT‐3′; anUisense: 5′‐ UUCCGAAUACUUGUCCAGCUCTT‐3′). Nontargeting *siRNA* duplex (Sangon Biotech, Negative control) served as the negative control. All microinjections were performed using a Narishige microinjector and completed within 30 min. The injected GV oocytes were arrested with IBMX for 12 h, allowing enough time for protein knockdown, followed by release into IBMX‐free M2 medium for further study.

### Time‐Lapse Live Imaging Experiments

The GV oocytes were cultured for 3 h at 37 °C to allow oocytes to enter the GVBD stage, and then oocytes were cultured in M2 medium containing Hoechst‐33342 (to label DNA, b2261, Sigma‐Aldrich) and SiR‐Tubulin (to image tubulin, CY‐SC002, Cytoskeleton). The live oocytes were imaged using the PerkinElmer precisely Ultra VIEW VOX Confocal Imaging System (PerkinElmer, Waltham, MA, USA).^[^ ^78^ ^]^ The imaging process took place in a controlled environment with a 5% CO_2_ atmosphere at 37 °C.

### Western Blot Analysis

A total of 200 mouse oocytes were collected and transferred to 10 µL 2%SDS sample buffer and boiled for 10 min at 95 °C for subsequent immunoblotting. To prepare cell protein extracts, cell was suspended in cold RIPA buffer (R0010, Solarbio) supplemented with a protein inhibitor cocktail (Roche Diagnostics, 04693116001, Rotkreuz, Switzerland) and 1 mm phenylmethyl sulfonyl fluoride (PMSF, 0754, Amresco). After homogenization and transient sonication, cell extracts were incubated on ice for 30 min. Then the samples were centrifuged at 12 000×rpm for 15 min at 4 °C. The supernatant was transferred to a new tube for immunoblotting. Protein lysates were separated via SDS‐PAGE and electro‐transferred to a nitrocellulose membrane. The membranes were blocked in PBS containing 5% skimmed milk, for 1 h at room temperature, followed by incubation overnight at 4 °C with primary antibody and incubated with secondary antibody for 1 h at room temperature. Finally, the membranes were washed three times in PBS buffer and scanned using an ODYSSEY Sa Infrared Imaging System (LI‐COR Biosciences, Lincoln, NE, USA).

### Cell Culture and Plasmid Transfection

HEK293T cells were grown in DMEM (Invitrogen) supplemented with 10% fetal bovine serum (Hyclone) and 1% penicillin‐streptomycin solution (Gibco) at 37 °C in a 5% CO_2_ incubator. cDNAs encoding mouse RNF20, RNF40, TPM3, RBBP4, RBBP7, UBASH3B, and H2Bub were subcloned into pCS2plus vector with MYC tag or pRK vector with FLAG tag for subsequent in vitro transcription and immunoprecipitation. HEK293T and Hela cells were transfected with plasmids by Lipofectamine 2000 (Invitrogen) or LipoMax DNA transfection reagent (Sudgen Biotechnology, China) according to the manufacturer's instructions.

### Co‐Immunoprecipitation

At 48 h of transfection, cells were lysed in lysis buffer (50 mm Tris‐HCl, pH 7.5, 150 mm NaCl, 10% glycerol, and 0.5% NP‐40, protease inhibitors were added before use). After sonication and high‐speed centrifugation of cell lysates, the supernatant was incubated with primary antibody overnight at 4 °C and then incubated with protein A‐Sepharose (GE, 17‐1279‐03) for 2 h at 4 °C. Thereafter, the precipitants were washed three times with IP buffer (20 mm Tris, pH 7.4, 2 mm EGTA, and 1% NP‐40), and bound proteins were analyzed by immunoblotting.

### Protein Purification and In Vitro Ubiquitination

For purification of GST‐RNF20/His‐RNF40, GST‐RNF20C922S/His‐RNF40, GST‐RNF20△RING/His‐RNF40, cDNA encoding mouse RNF20 and RNF40 were cloned into pGEX‐4t‐1 and pET28a respectively. RNF20 mutants were created using KOD‐Plus‐Neo (KOD‐401) enzyme. Then, pET28a‐RNF40 and pGEX‐4t‐1‐RNF20 or RNF20 mutant were transformed into BL21 (DE3) cells and cultured them in Terrific Broth at 37 °C. The cells were transferred to a low‐temperature shaker (16 °C) and induced with 0.25 mm isopropyl‐D‐thiogalactoside (IPTG, 1758‐1400‐100 g) for 16 h. Subsequently, the cells were harvested, resuspended in His lysis buffer (20 mm Tris, pH 7.4, 500 mm NaCl, 10 mm imidazole, 10% glycerol), and lysed using sonication. The supernatant was collected after high‐speed centrifugation and incubated with Ni Sepharose 6 Fast Flow (GE Healthcare, Marlborough, MA) at 4 °C for 2 h. The beads were then washed, and the protein was eluted using the lysis buffer supplemented with 300 mm imidazole. in vitro ubiquitination assay was performed as described previously.^[^ ^79^ ^]^ Briefly, the ubiquitination experiments were conducted as follows: nucleosomal histones (1ug, Active Motif, 31466), E1 (60 nm), E2 (200 nm) and RNF20/40 (200 nm) or RNF20 C922S/40 (200 nm), RNF20△RING/40 (200 nm) were incubated with FLAG‐Ub (10 nm) at 37 °C for 1.5 h in a buffer containing 25 mm Tris‐HCl (pH 7.4), 2 mm ATP and 0.1 mm DTT. The resulting products were separated by SDS‐PAGE and analyzed by western blotting.

### Statistical Analysis

Statistical analyses were conducted using GraphPad PRISM version 9. All experiments were repeated at least three times and all data were expressed as mean ± SEM or mean ± SD. For box plots, the central line and plus sign indicate the median and mean, respectively. The rectangle box represents the minimum to maximum values, with each individual value as point in the graph. The statistical significance of the differences between the mean values for the different genotypes was measured by a two‐tailed unpaired student's *t*‐test. The data were considered statistically significant when the *p*‐value was less than 0.05 (*), 0.01 (**), or 0.001 (***).

## Conflict of Interest

The authors declare that they have no conflict of interest.

## Author Contributions

L.W., C.L., and L.L. contributed equally to this work. L.W., C.L., and L.L. performed conceptualization, investigation, data curation, validation, project administration, methodology, formal analysis, and wrote the original draft. H.W., and W.W. performed investigation and methodology. L.W., Q.Z., Y.C., and T.M. performed investigation, methodology and formal analysis. R.J. acquired resources and did supervision. Z.W., Q.S., and W.L. performed conceptualization, supervision, acquired resources and funding, wrote the original draft and reviewed and edited the final manuscript.

## Supporting information

Supporting Information

## Data Availability

The data that support the findings of this study are available from the corresponding author upon reasonable request.
