# Supplementary material for: RNF20 Regulates Oocyte Meiotic Spindle Assembly by Recruiting TPM3 to Centromeres and Spindle Poles
Source: Adv Sci (Weinh). 2024 Jan 19;11(13):2306986. doi: 10.1002/advs.202306986 (PMC10987117; doi:10.1002/advs.202306986)
Supplement: Supplementary file 1 — Supporting Information [file ADVS-11-2306986-s001.pdf]

ADVANCED SCIENCE
Open Access

Supporting Information

for *Adv. Sci.*, DOI 10.1002/advs.202306986

RNF20 Regulates Oocyte Meiotic Spindle Assembly by Recruiting TPM3 to Centromeres and Spindle Poles

Liying Wang, Chao Liu, Li Li, Huafang Wei, Wei Wei, Qiuxing Zhou, Yinghong Chen, Tie-Gang Meng, Renjie Jiao, Zhen-Bo Wang, Qing-Yuan Sun* and Wei Li**

RNF20 Regulates Oocyte Meiotic Spindle Assembly by Recruiting TPM3 to Centromeres and Spindle Poles

Liying Wang[1,†], Chao Liu[1,2,5,†], Li Li[1,†], Huafang Wei[1], Wei Wei[1], Qiuxing Zhou[1], Yinghong Chen[2,5], Tie-Gang Meng[3], Renjie Jiao[4], Zhen-Bo Wang[2,5,*], Qing-Yuan Sun[3,*], Wei Li[1,2,5,*]

Supplementary Materials:

This file includes:

Figure S1 to S5

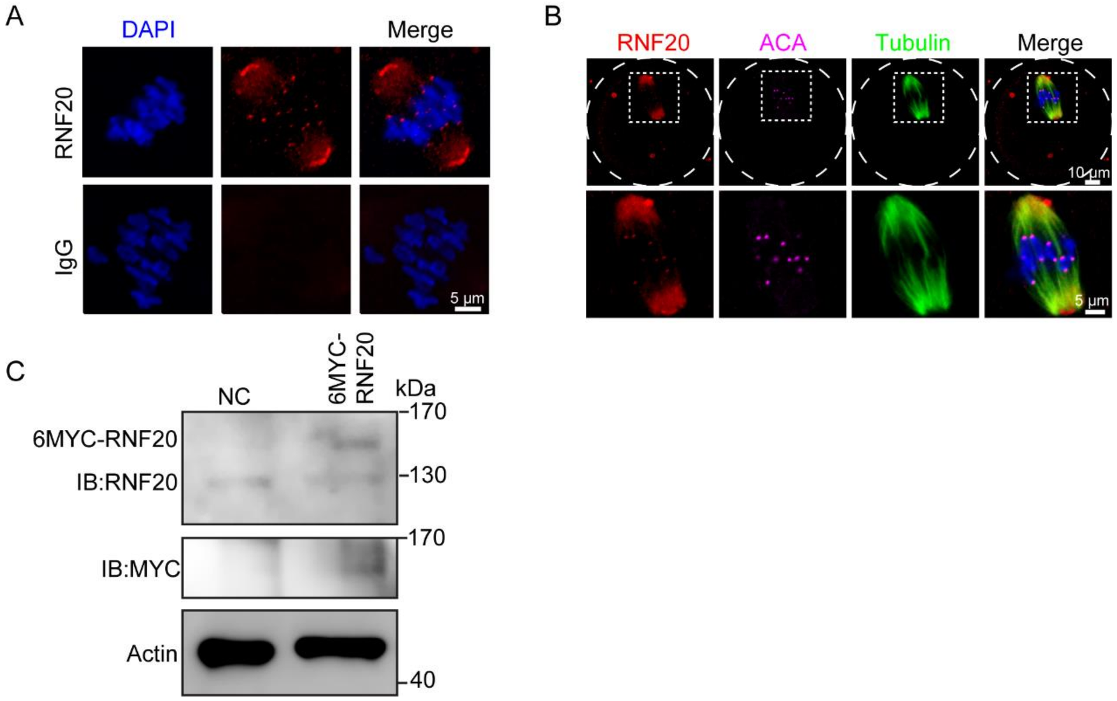

Figure S1. RNF20 is localized at centromeres and spindle poles

(A) RNF20 (red) is localized at centromeres and spindle poles, IgG served as a negative control.

(B) Immunofluorescence showing localization of RNF20 (red). ACA (magenta) served as a marker for centromere.

(C) Western blotting results showing protein expression after injecting MYC-RNF20 mRNAs. Total proteins from100 injected oocytes were loaded in each lane. The blots were probed with anti-MYC, anti-RNF20 and anti-Actin antibodies. Actin served as a loading control. The same amount of RNase-free water was injected as negative control.

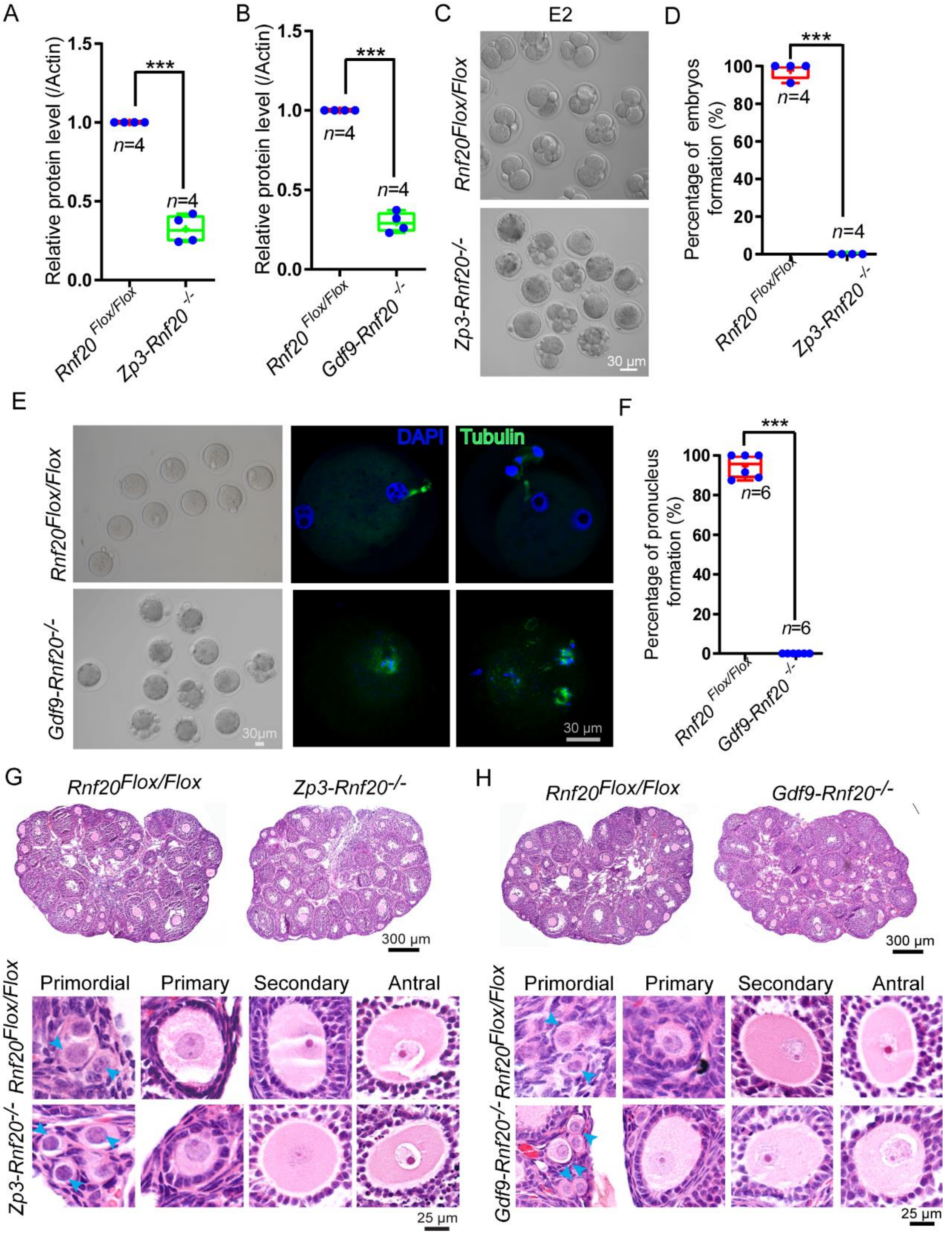

Figure S2. RNF20 is required for fertilization and early embryonic development

(A) Odyssey-based quantification of protein levels in Figure 2A. (n = 4 independent experiments). Data are presented as mean ± SEM. $^{***}p < 0.001$. Statistical analysis was performed with two-tailed unpaired Student's t test.

(B) Odyssey-based quantification of protein levels in Figure 2B. (n = 4 independent experiments). Data are presented as mean ± SEM. $^{***}p < 0.001$. Statistical analysis was performed with two-tailed unpaired Student's t test.

(C) Representative images of embryos derived from *Rnf20$^{Flox/Flox}$* and *Zp3-Rnf20$^{-/-}$* females.

(D) Percentage of embryos formation in C. *Rnf20$^{Flox/Flox}$*, 97.75% ± 2.25; *Zp3-Rnf20$^{-/-}$*, 0.00% ± 0.00.

Data are presented as mean ± SEM. ***$p < 0.001$. n = 4 mice. More than 50 embryos were observed in each experimental group with similar results. Statistical analysis was performed with two-tailed unpaired Student's t test.

(E) Representative images of embryos derived from *Rnf20*$^{Flox/Flox}$ and *Gdf9-Rnf20*$^{-/-}$ females. n = 6 mice for zygote stage. Immunofluorescence showing pronuclear stage (PN) zygotes of *Rnf20*$^{Flox/Flox}$ and *Gdf9-Rnf20*$^{-/-}$ mice.

(F) Percentage of pronucleus formation of *Rnf20*$^{Flox/Flox}$, 94.67% ± 2.45% (n = 6); *Gdf9-Rnf20*$^{-/-}$, 0.00% ± 0.00% (n = 6). Data are presented as mean ± SEM. ***$p < 0.001$. Statistical analysis was performed with two-tailed unpaired Student's t test.

(G) H&E staining of ovaries from 3-week-old *Rnf20*$^{Flox/Flox}$ and *Zp3-Rnf20*$^{-/-}$ mice. The arrows indicate primordial follicles.

(H) H&E staining of ovaries from 3-week-old *Rnf20*$^{Flox/Flox}$ and *Gdf9-Rnf20*$^{-/-}$ mice. The arrows indicate primordial follicles.

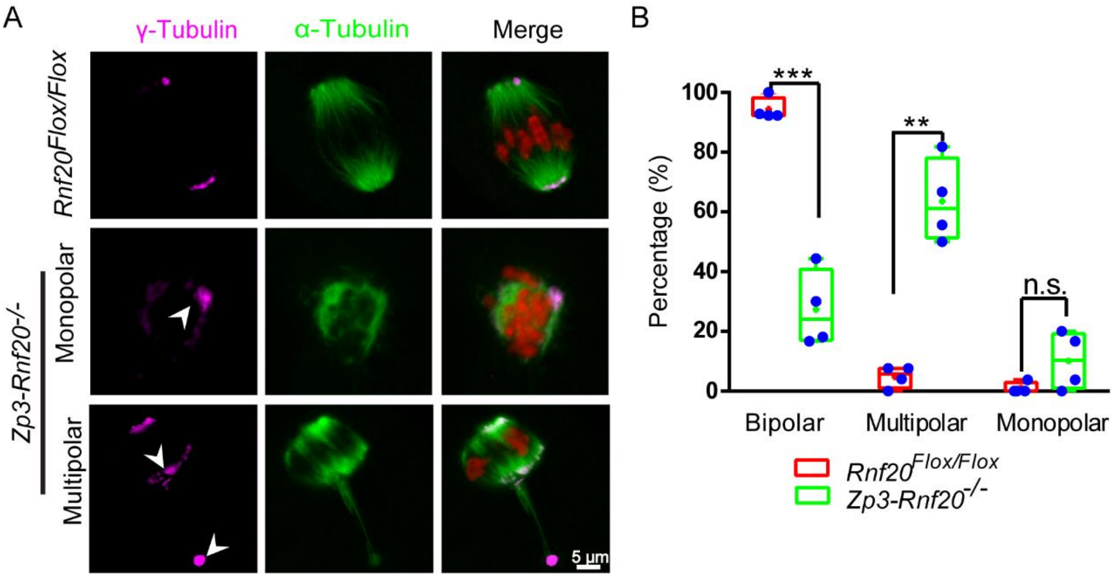

Figure S3. Lack of RNF20 perturbs bipolar spindle assembly

(A) Representative images of spindle morphology in WT and RNF20-depleted oocytes. Metaphase I oocytes were fixed and stained with anti-α-tubulin antibody (green) to detect spindle microtubules and anti-γ-tubulin antibody (magenta) to detect spindle poles. Arrows indicate spindle poles.

(B) Percentage of bipolar, multipolar, and monopolar in *Rnf20*$^{Flox/Flox}$ and *Zp3-Rnf20*$^{-/-}$ oocytes. Data are presented as mean ± SEM. n.s., non-significant. **$p < 0.01$ and ***$p < 0.001$. Statistical analysis was performed with two-tailed unpaired Student's t test.

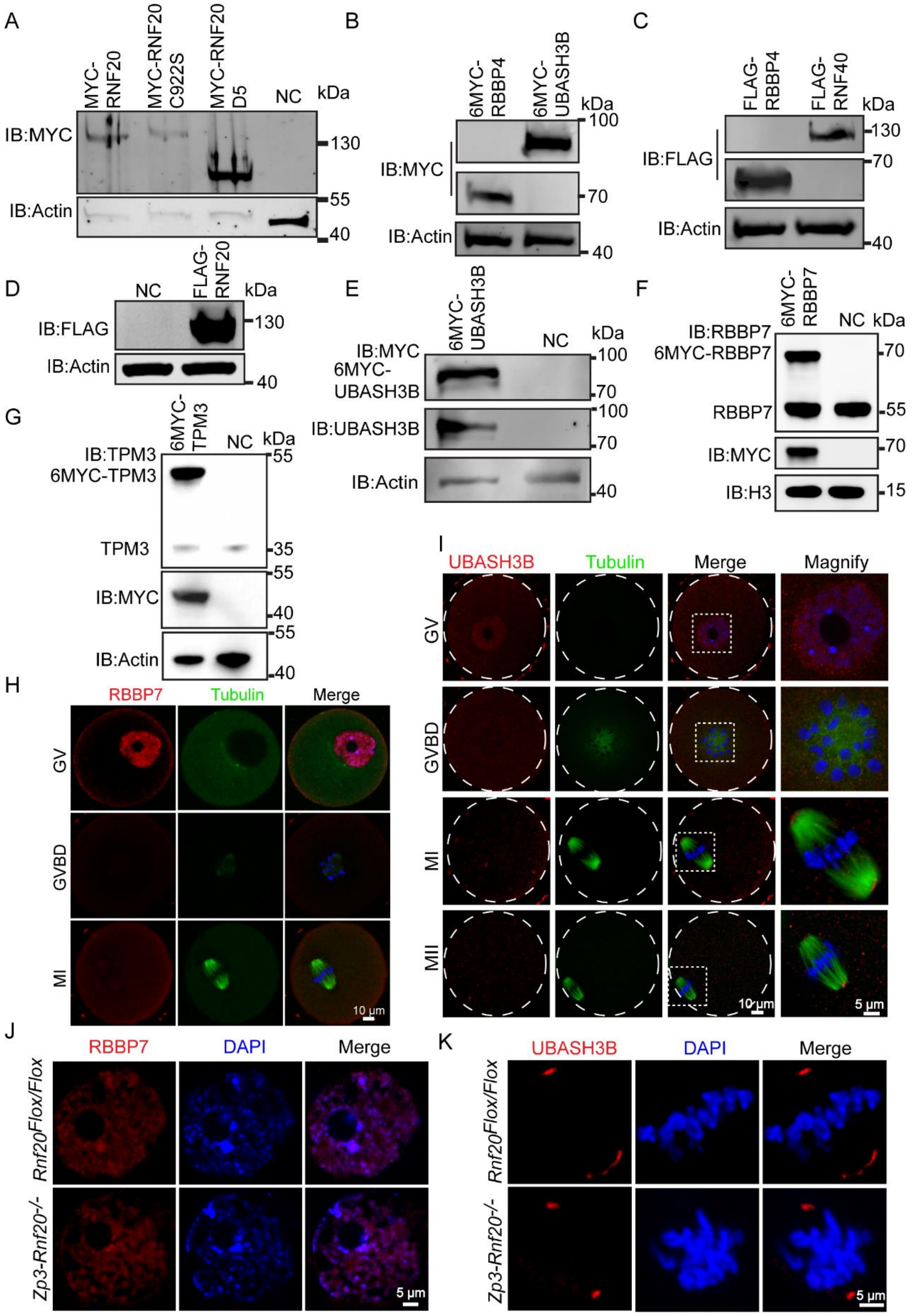

Figure S4. Localization of RBBP7 and UBASH3B during mouse oocyte meiotic maturation

(A) Western blotting results showing protein expression after injecting MYC-RNF20 mRNA, MYC-RNF20 C922S mRNA and MYC-RNF20 D5 mRNA. The blots were probed with anti-MYC and anti- Actin antibodies. The same amount of RNase-free water was injected as negative control.

(B-D) Representative images of 6MYC-RBBP4, 6MYC-UBASH3B, FLAG-RNF40 and FLAG-RNF20

vectors expression in HEK293T cells.

(E-G) Representative images demonstrating the specificity of the anti-UBASH3B, anti-RBBP7 and anti-TPM3 antibodies in HEK293T cells

(H) Representative images showing RBBP7 localization in GV, GVBD and MI stages during meiosis.

(I) Representative images showing UBASH3B localization in GV, GVBD, MI and MII stages during meiosis.

(J) Representative images showing RBBP7 localization in WT and RNF20-depleted oocytes. GV stage oocytes were immunolabeled with anti-RBBP7 antibody (red) and counterstained with DAPI (blue).

(K) Representative images showing UBASH3B localization in WT and RNF20-depleted oocytes. MI stage oocytes were immunolabeled with anti-UBASH3B antibody (red) and counterstained with DAPI (blue).

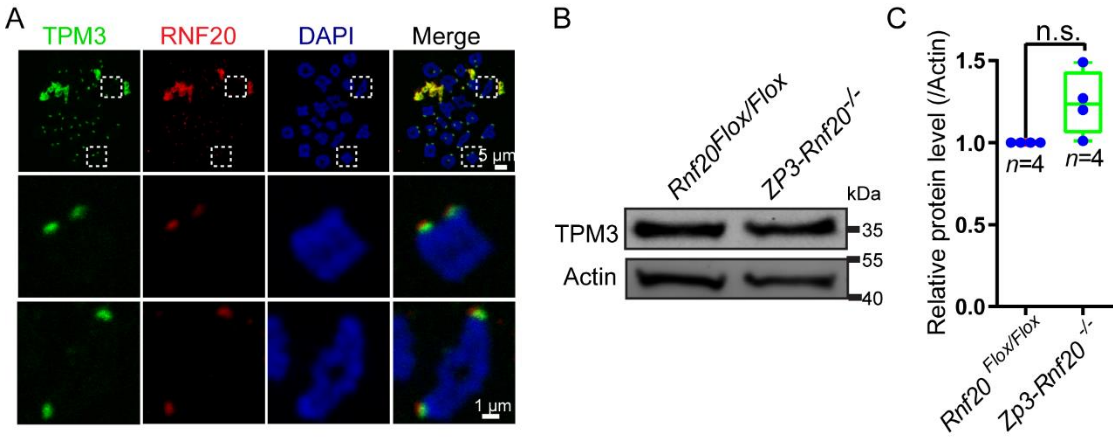

Figure S5. Localization of TPM3 in oocytes

(A) Chromosome spreading showing co-localization of RNF20 and TPM3.

(B) Western blotting showing the expression levels of TPM3 in WT and RNF20-depleted oocytes. Actin served as a loading control.

(C) Relative protein levels of TPM3 in *Rnf20*$^{Flox/Flox}$ and *Zp3-Rnf20*$^{-/-}$ oocytes. (n = 4 independent experiments). Data are presented as mean ± SEM. n.s., non-significant. Statistical analysis was performed with two-tailed unpaired Student's t test.
